# Toll-Like Receptor 4 Activation Prevents Rat Cardiac Fibroblast Death Induced by Simulated Ischemia/Reperfusion

**DOI:** 10.3389/fcvm.2021.660197

**Published:** 2021-06-08

**Authors:** Pablo Parra-Flores, Jenaro Espitia-Corredor, Claudio Espinoza-Pérez, Cristian Queirolo, Pedro Ayala, Francisca Brüggendieck, Aimee Salas-Hernández, Viviana Pardo-Jiménez, Guillermo Díaz-Araya

**Affiliations:** ^1^Laboratorio de Farmacología Molecular, Departamento de Química Farmacológica y Toxicológica, Facultad de Ciencias Químicas y Farmacéuticas, Universidad de Chile, Santiago, Chile; ^2^Department of Pharmacology, Faculty of Medicine, Instituto de Investigación Sanitaria Hospital Universitario La Paz, Universidad Autónoma de Madrid, Madrid, Spain; ^3^Departamento de Enfermedades Respiratorias, Pontificia Universidad Católica de Chile, Santiago, Chile; ^4^Department of Pharmacology, Toxicology and Pharmacodependence, Pharmacy Faculty, University of Costa Rica, San José, Costa Rica; ^5^Advanced Center for Chronic Diseases, Facultad de Ciencias Químicas y Farmacéuticas, Universidad de Chile, Santiago, Chile

**Keywords:** cardiac fibroblasts, ischemia/reperfusion, TLR4, LPS, apoptosis

## Abstract

Death of cardiac fibroblasts (CFs) by ischemia/reperfusion (I/R) has major implications for cardiac wound healing. In *in vivo* models of myocardial infarction, toll-like receptor 4 (TLR4) activation has been reported as a cardioprotector; however, it remains unknown whether TLR4 activation can prevent CF death triggered by simulated I/R (sI/R). In this study, we analyzed TLR4 activation in neonate CFs exposed to an *in vitro* model of sI/R and explored the participation of the pro-survival kinases Akt and ERK1/2. Simulated ischemia was performed in a free oxygen chamber in an ischemic medium, whereas reperfusion was carried out in normal culture conditions. Cell viability was analyzed by trypan blue exclusion and the MTT assay. Necrotic and apoptotic cell populations were evaluated by flow cytometry. Protein levels of phosphorylated forms of Akt and ERK1/2 were analyzed by Western blot. We showed that sI/R triggers CF death by necrosis and apoptosis. In CFs exposed only to simulated ischemia or only to sI/R, blockade of the TLR4 with TAK-242 further reduced cell viability and the activation of Akt and ERK1/2. Preconditioning with lipopolysaccharide (LPS) or treatment with LPS in ischemia or reperfusion was not protective. However, LPS incubation during both ischemia and reperfusion periods prevented CF viability loss induced by sI/R. Furthermore, LPS treatment reduced the sub-G1 population, but not necrosis of CFs exposed to sI/R. On the other hand, the protective effects exhibited by LPS were abolished when TLR4 was blocked and Akt and ERK1/2 were inhibited. In conclusion, our results suggest that TLR4 activation protects CFs from apoptosis induced by sI/R through the activation of Akt and ERK1/2 signaling pathways.

## Introduction

Cardiovascular diseases (CVDs) have remained as the main cause of death worldwide during the last decades (1). Reperfusion therapy is the primary clinical management in patients with myocardial infarction to restore the normal coronary circulation in the cardiac ischemic tissue and limit the extent of necrosis (2). However, the intracellular biochemical changes during ischemia, followed by the abrupt reperfusion, induce additional cell death associated with the generation of reactive oxygen species (ROS), which produce oxidative modification of macromolecules (proteins, lipids, carbohydrates, and nucleic acids) and trigger apoptotic pathways (3, 4). Currently, many successful cardioprotective strategies against myocardial ischemia/reperfusion (MIR) have been reported in experimental animal models, but translation to clinical practice has proven difficult due to the complexity of MIR (5). Combinations of therapies with synergistic effects, as well as protection of all cardiac cell types—and not just cardiomyocytes—are believed to be essential to achieve cardioprotection in the clinical arena (5, 6).

Cardiac fibroblasts (CFs) are involved in the homeostasis of extracellular matrix (ECM) proteins in normal cardiac tissue. In addition, when an injury occurs in the heart, CFs initiate an inflammation response by secreting many cytokines and growth factors and differentiate into cardiac myofibroblasts (CMFs) to produce collagen, leading to wound healing and scar formation (6, 7). It is well-known that simulated ischemia/reperfusion (sI/R) can produce deleterious effects on CF viability; however, strategies to prevent sI/R injury have been poorly evaluated. This is an important topic since CFs participates in wound healing processes; therefore, viability protection is of utmost importance for their protection (8–11).

Toll-like receptors (TLRs) are a family of receptors present in immune system cells, which recognize and react to highly conserved motifs known as damage-associated molecular patterns (DAMPs; “alarmins”) and pathogen-associated molecular patterns [PAMPs, like lipopolysaccharide (LPS)], and mediate the immune innate response and inflammatory process after tissue injury, remodeling, stress, infection, and other situations. In addition, TLRs are highly expressed in vascular cells and cardiac tissue (12, 13), and TLR4 is one of the most studied isoforms. Reports in rat CFs have indicated that TLR4 activation by LPS activates Akt, ERK1/2, and NF-kB signaling pathways. This activation promotes a strong proinflammatory response characterized by the release of cytokines (IL1-β and TNF-α) and chemokines (MCP-1), increased expression of the cellular adhesion molecules ICAM-1 and VCAM-1 and the B1 bradykinin receptor, and prevention of CF-to-CMF differentiation (14–17). Furthermore, other studies in mice have reported that activation of TLR4 by LPS produces a cardioprotective response through the PI3K/Akt pathway against MIR (18, 19).

At the cardiac cellular level, it is well-known that sI/R triggers CF necrosis and apoptosis (10), and we recently showed that the use of antioxidants prevents CF death (11). Therefore, the search of alternative therapies to decrease CF death and, consequently, to improve the wound healing process has become relevant; however, until now, it is unknown whether TLR4 activation can produce a cytoprotective effect in CFs exposed to sI/R conditions. Thus, the aim of this study was to determine whether TLR4 activation (by LPS as a specific agonist) protects CFs from apoptotic death triggered by sI/R and to elucidate the participation of the Akt and ERK1/2 signaling pathways, which are recognized as part of reperfusion injury salvage kinase (RISK). The RISK pathway corresponds to the activation of two parallel cascades: PI3K-Akt and MEK1-ERK1/2, a group of pro-survival protein kinases which that cardioprotection when activated specifically at the time of reperfusion (20).

## Methods and Materials

### Reagents

Dulbecco's modified Eagle's medium F12 (DMEM-F12) and Collagenase Type II (powder) were obtained from Thermo Fisher Scientific (Waltham, MA, USA). Fetal bovine serum (FBS), trypsin ethylenediaminetetraacetic acid (EDTA; 0.5%), EDTA 0.2% (10 × solution), penicillin–streptomycin–amphotericin B solution, and trypan blue (0.5%) solutions were obtained from Biological Industries (Cromwell, CT, USA). The MEK1/2-ERK1/2 inhibitor PD98059, pancreatin from porcine pancreas, RNAse A, propidium iodide (PI), Bradford reagent, radioimmunoprecipitation assay (RIPA) lysis and extraction buffers, Halt Protease Inhibitor Cocktail (100×), Halt Phosphatase Inhibitor Cocktail, and enhanced chemiluminescence (ECL) western blotting detection reagents were obtained from Sigma-Aldrich Corp. (St. Louis, MI, USA). The MTT Assay Kit and Prestained Protein Ladder-Broad molecular weight (10–245 kDa) were obtained from Abcam (Cambridge, MA, USA). Nitrogen gas (N_2_) cylinders were obtained from Linde Group (Santiago, Chile). Primary antibodies for phospho-ERK1/2 (p-ERK1/2), phospho-Akt (p-Akt), glyceraldehyde 3-phosphate dehydrogenase (GAPDH), and the secondary antibodies anti-rabbit IgG and anti-mouse IgG conjugated with horseradish peroxidase (HRP) were obtained from Cell Signaling Technology (Danvers, MA, USA). All plastic materials were obtained from Corning Incorporated (Corning, NY, USA). The PI3K-Akt inhibitor LY294002 was obtained from Cayman Chemicals (Ann Arbor, MI, USA). LPS-EB, LPS-EB ultrapure, and TAK-242 (inhibitor of TLR4 signaling) were obtained from InvivoGen (San Diego, CA, USA). All inorganic salt products and methanol were obtained from Merck (Darmstadt, Germany).

### Isolation and Culture of Cardiac Fibroblasts

Sprague–Dawley neonate rats (1- to 3-day-old) were obtained from the Animal Breeding Facility of the Faculty of Chemical and Pharmaceutical Sciences at University of Chile. All studies followed the Guide for the Care and Use of Laboratory Animals published by the US National Institutes of Health (NIH Publication No. 85–23, revised 1996), and experimental protocols were approved by our Institutional Ethics Review Committee. CFs were isolated as previously described (10). In a sterile zone, rats were swiftly decapitated, and their hearts were removed. In brief, ventricles were minced and digested in a solution that contained collagenase (0.05%) and pancreatin (0.05%). The digestion homogenized product was pre-cultured on 100-mm-diameter plastic plates, with culture medium containing FBS (10%) and penicillin–streptomycin–amphotericin B, and kept in an incubator with O_2_ (95%) and CO_2_ (5%) at 37°C. The CFs adhered differentially to plastic, allowing their isolation from cardiomyocytes. After 2 h, the culture medium was replaced with DMEM-F12 containing 10% FBS medium, and CFs were left to proliferate to confluence (3–5 days) in the same environment conditions. The medium was then replaced with DMEM-F12 containing FBS (3%) and penicillin–streptomycin–amphotericin B. Cells underwent up to a maximum of two passages, and detachment was performed using trypsin EDTA (0.5%) and EDTA 0.2% (1×), followed by protease inhibition with DMEM-F12 containing FBS (10%). CFs were then collected and seeded on plastic plates in DMEM-F12 medium without FBS for 24 h before the experiments.

### Protocol of *in vitro*-Simulated Ischemia/Reperfusion and Stimulation With Lipopolysaccharide/Inhibitors

**Figure 1:**
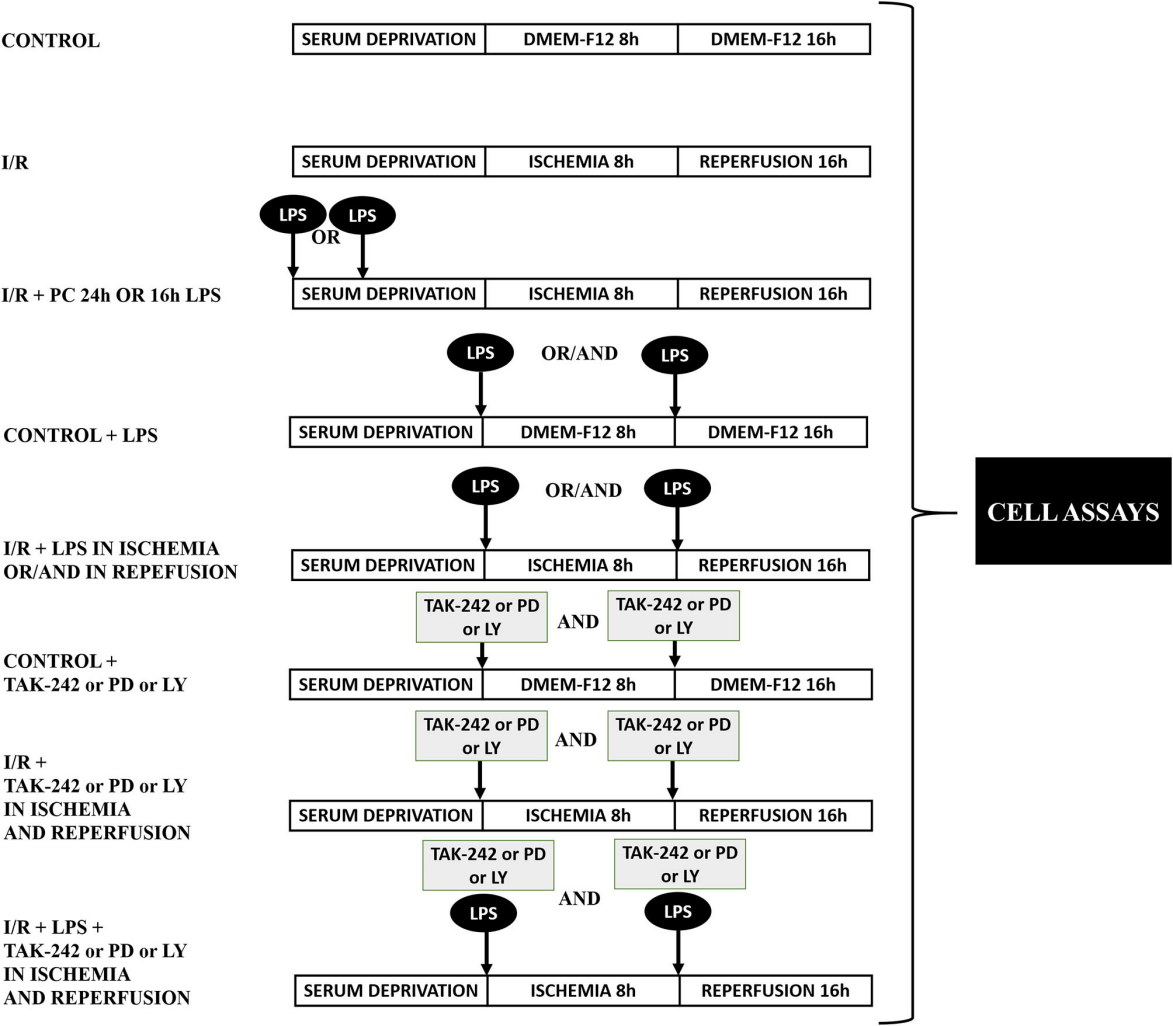
Protocol of experimental design. Representative diagram of the experimental groups in the biological assays.

After 24 h without serum, CFs were washed with phosphate-buffered saline (PBS) before the ischemic protocol. The cells were exposed to a balanced salt solution (ischemic medium) with pH 6.2: NaCl 139 mM, KCl 12 mM, MgCl_2_ 0.5 mM, CaCl_2_ 0.9 mM, HEPES 5 mM, lactic acid 20 mM, and 2-deoxy-d-glucose 10 mM. Hypoxia was achieved in a customized chamber with N_2_ environment at 37°C for 8 h. A reperfusion protocol was developed by replacing the ischemic medium with DMEM-F12 in a 95% air/5% CO_2_ incubator (37°C) for 16 h. Control cells were incubated under normoxic conditions in DMEM-F12 medium and kept in the incubator with O_2_ (95%) and CO_2_ (5%) at 37°C for the exact duration of simulated ischemia and sI/R experiments. CFs were stimulated with LPS (1 μg/ml) under the following conditions: (i) 24 and 16 h before ischemia; (ii) at the onset of ischemia or reperfusion; and (iii) at the onset of ischemia and consecutive reperfusion, depending on the experimental conditions and with the respective controls. TAK-242 (1 μM), LY294002 (10 μM), and PD98059 (25 μM) were added in the presence/absence of LPS, depending on the experimental conditions and with the respective controls. Figure 1 shows graphically the different experimental protocols used in the biological assays.

### Cell Viability by Trypan Blue Exclusion

CFs were plated at a density of 156 cells/mm^2^ on 35-mm plastic dishes and stimulated under the conditions indicated for each experiment. After 16 h of simulated reperfusion, cells were washed two times with PBS and treated with trypsin EDTA (0.5%) and EDTA 0.2% (1×) to detach cells, followed by administration of FBS (10%) to induce inactivation. After detachment, aliquots of 20 μl of sample plus 20 μl of trypan blue (0.5%) reagent were homogenized, and then 8 μl was transferred to a Neubauer chamber (Paul Marienfeld GmbH & Co. KG, Lauda-Königshofen, Germany) to count viable cells (unstained) using optic microscopy. Experiments were performed in duplicate and repeated six times. At least 1,000 cells/ml was counted in each sample.

### Cell Viability by the MTT Assay

CFs at a density of 156 cells/mm^2^ were plated on 35-mm plastic dishes in DMEM-F12 medium and stimulated under the conditions indicated for each experiment. At the end of reperfusion, cells were washed with PBS and then kept in DMEM-F12 medium. An aliquot of filtered MTT solution (5 mg/ml) was added in a 1:10 ratio, and the cells were incubated for 2.5 h in a 95% air/5% CO_2_ incubator (37°C). The cells were then washed with PBS, and an aliquot of isopropanol (1:10 ratio) was added to solubilize the MTT crystals. The solubilized extracts were seeded on 96-well plates with transparent bottom, and the absorbance at 570 nm was registered (Epoch UV-Vis Spectrophotometer, BioTek).

### Western Blot Analysis

For protein content analysis, CFs were seeded at a density of 106 cells/mm^2^ on 60-mm plastic dishes in DMEM-F12 medium and were stimulated under the conditions indicated for each experiment. At the end of reperfusion, cells were washed with cold PBS and then treated with RIPA lysis buffer containing protease and phosphatase inhibitors. Samples were centrifuged at 252 × g for 10 min at 4°C, and supernatants were collected. The total protein concentration of samples was determined by spectrophotometry using the Bradford assay reagent, by reading the absorbance at 595 nm (Epoch UV-Vis Spectrophotometer, BioTek). Twenty-five micrograms of total protein extracts (with charge buffer) was separated by 15% acrylamide/bis-acrylamide sodium dodecyl sulfate–polyacrylamide gel electrophoresis (SDS-PAGE) at constant 90 V for 1.5 h. Proteins were then electro-transferred to a nitrocellulose membrane for 1.5 h, at constant 0.4 A. The membranes were blocked with non-fat milk 5% (w/v) for 1 h and incubated overnight at 4°C with primary antibodies against p-ERK1/2, p-Akt (dilution 1:1,000 for both) or GAPDH (dilution 1:5,000). The membranes were then washed and incubated for 1.5 h at room temperature with anti-rabbit IgG or anti-mouse IgG conjugated with HRP (dilution 1:5,000) secondary antibodies. After being washed, membranes were exposed to the ECL reagent and revealed by two methods: (i) C-DiGit Chemiluminescent Western Blot Scanner (LI-COR Biosciences, Lincoln, NE, USA), wherein images and blots were analyzed and quantified using the Image Studio™ software (LI-COR Biosciences, Lincoln, NE, USA), or (ii) BioMax film in a dark room, wherein films were scanned and blots were quantified with the ImageJ software (LI-COR Biosciences, Lincoln, NE, USA).

### Necrosis Determination by Flow Cytometry

CFs were seeded with a density of 106 cells/mm^2^ on 60-mm plastic dishes and were stimulated under the conditions indicated for each experiment. At the end of reperfusion, dead cells were collected as a pellet from the medium after centrifuging at 252 × g for 5 min and kept them on ice. Live cells on plates were detached with trypsin EDTA (0.5%) and EDTA 0.2% (1×) and mixed with the dead cell pellet. Subsequently, PI (1 mg/ml) was added to the cell mixture. Cell necrosis was assessed by flow cytometry in a BD FACSCantoA (Becton Dickinson & Company, Franklin Lakes, NJ, USA). A total of 5,000 cells/sample were analyzed.

### Determination of the Sub-G1 Population by Flow Cytometry

CFs were seeded at a density of 106 cells/mm^2^ on 60-mm plastic dishes and were stimulated under the conditions indicated for each experiment. At the end of reperfusion, dead cells of the medium and living cells on the plates were collected according to the same protocol described in the Necrosis Determination by Flow Cytometry section. Cold methanol was added to the mixture of live and dead cells to permeabilize their cell membranes, overnight at −20°C. Later, RNAse (0.1 mg/ml) was added to the samples for 1 h at room temperature. Finally, PI (1 mg/ml) was added to the cells, and apoptosis was determined using a BD FACSCanto (Becton Dickinson & Company, Franklin Lakes, NJ, USA) flow cytometer. PI marks condensed chromatin and/or fragmented DNA in apoptotic bodies, giving a low-intensity signal (sub-G1 population), under the prominent G1 signal of living cells with integral DNA. A total of 5,000 cells/sample were analyzed.

### Statistical Analysis

All data are presented as mean ± SEM of at least four to six independent experiments and were analyzed using the version 5.01 of the GraphPad Prism software (GraphPad, San Diego, CA, USA). The differences between experimental groups were evaluated by one-way ANOVA, followed by the Tukey post-test. Statistical significance was accepted at *p* < 0.05.

## Results

### Lipopolysaccharide Treatment Administered at the Beginning of Ischemia and Reperfusion Increased Cell Viability and Reduced Apoptosis of Cardiac Fibroblasts

**Figure 2:**
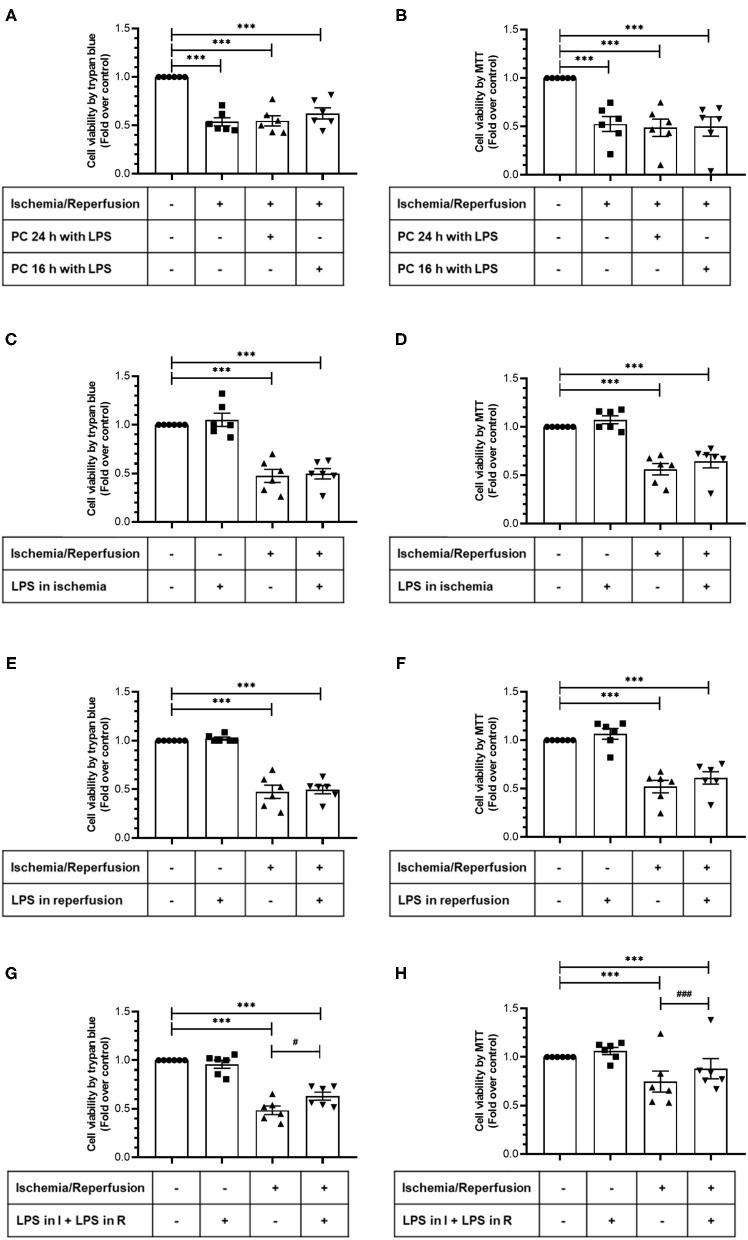
Lipopolysaccharide (LPS) increases the viability of cardiac fibroblasts exposed to simulated ischemia/reperfusion (sI/R). Cardiac fibroblasts were subjected to 8 h of ischemia and 16 h of reperfusion (sI/R). Cell viability was determined using the trypan blue exclusion test **(A,C,E,G)** or the MTT assay **(B,D,F,H)**. **(A,B)** Effects of preconditioning (PC) with LPS. Cardiac fibroblasts were pretreated with LPS (1 μg/ml) for 24 and 16 h before performing sI/R. **(C,D)** Effect of LPS only during ischemia. Cardiac fibroblasts were treated with LPS (1 μg/ml) during the 8 h of ischemia and then were perfused (16 h). **(E,F)** Effect of LPS only during reperfusion. Cardiac fibroblasts were treated with LPS (1 μg/ml) only during the 16 h of reperfusion. **(G,H)** Effect exerted by LPS during ischemia and reperfusion. Cardiac fibroblasts were treated with LPS (1 μg/ml) during the 8 h of ischemia and the 16 h of reperfusion. Results are expressed as mean ± SEM of six independent experiments. ***p < 0.001 vs. control; #*p* < 0.05 and ###*p* < 0.001 vs. sI/R.

**Figure 3:**
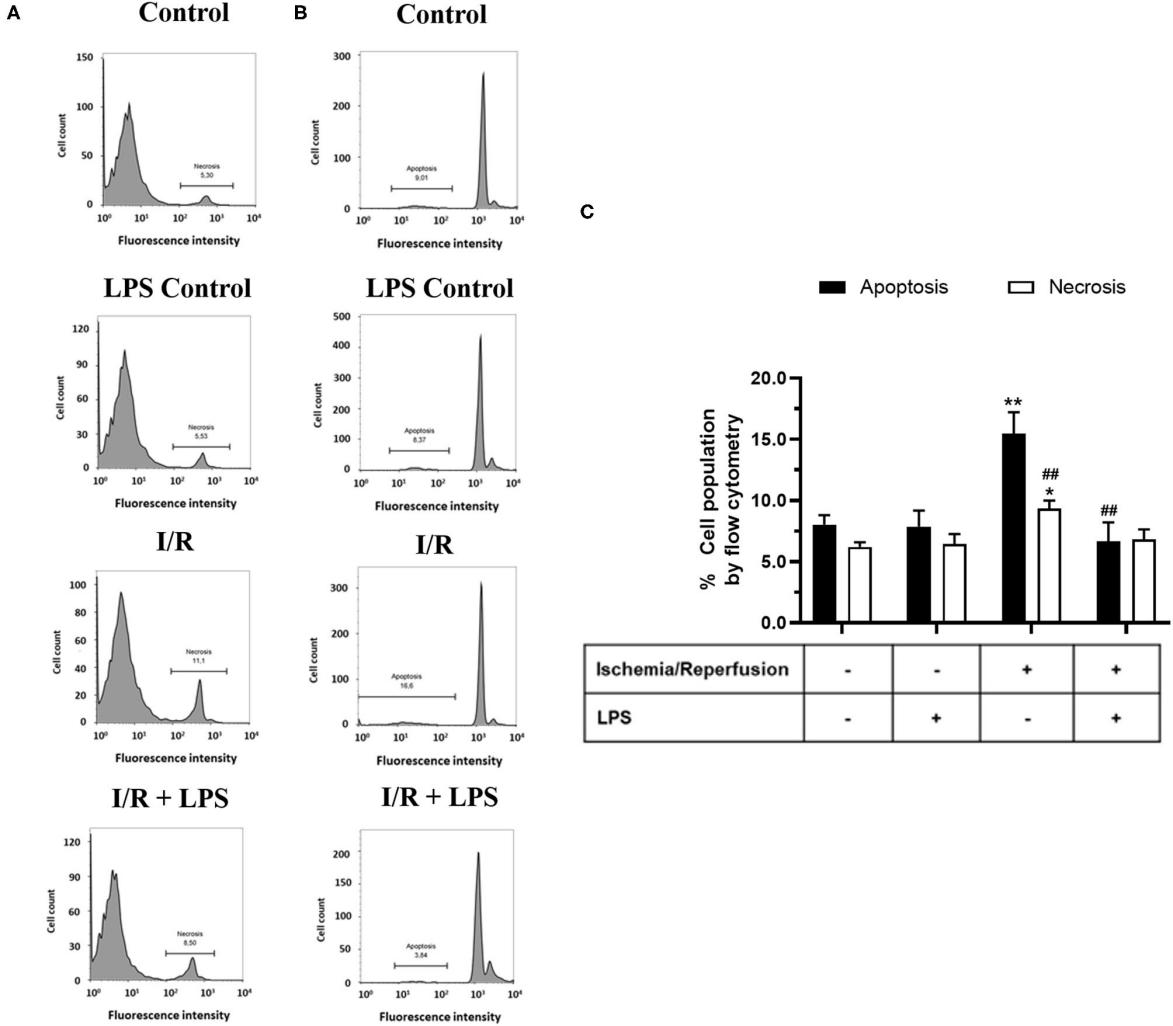
Lipopolysaccharide (LPS) reduces apoptosis induced by simulated ischemia/reperfusion (sI/R) in cardiac fibroblasts. Cardiac fibroblasts were exposed to 8 h of ischemia, followed by 16 h of reperfusion (sI/R). Cells were treated with LPS (1 μg/ml) at the onset of ischemia and at the start of reperfusion. **(A)** The percentage (%) of necrosis was quantified by flow cytometry using propidium iodide (left), with representative histograms of each experimental group and the graphical analysis on the right. **(B)** The percentage (%) of the sub-G1 population (apoptosis) was quantified by flow cytometry using propidium iodide (middle), with representative histograms of each experimental group and the **(C)** graphical analysis on the right panel. The results are expressed as mean ± SEM of five independent experiments. *p < 0.01 and **p < 0.01 vs. each population control, ##*p* < 0.01 vs. apoptosis population in sI/R.

In order to study the cytoprotective effects of LPS, we first evaluated different LPS treatments times in our model by subjecting neonatal rat CFs to 8 h of simulated ischemia, followed by 16 h of simulated reperfusion; and then we measured cell viability using both the trypan blue and MTT assays. Regarding the sensitivity of cardiac fibroblasts to hypoxia, the ischemia time of 8 h was chosen from previous studies carried out in our laboratory (10) in which it was shown that 8 h of ischemia is the minimum time of ischemia in which a statistically significant decrease in cell viability is not observed, while in longer times of ischemia, the loss of cell viability varies between 40% at 12 h and 85% at 24 h. The results show that cell viability did not increase with LPS treatment (1 μg/ml) in a preconditioning manner during 24 and 16 h, before performing sI/R (Figures 2A,B); at the beginning of simulated ischemia (Figures 2C,D); or at the beginning of simulated reperfusion (Figures 2E,F). However, when we administered LPS (1 μg/ml) at the beginning of both ischemia and reperfusion, cell viability increased when compared with untreated conditions (*p* < 0.05; Figures 2G,H). We then evaluated the effects of LPS treatment on cell death types (necrosis and apoptosis) induced by sI/R, using flow cytometry analysis with PI staining. The results indicate that CFs exposed to sI/R increase the percentage of cells in necrosis and apoptosis compared with control, the last one in a higher level than necrotic population. Second, the CFs exposed to sI/R and treated with LPS (1 μg/ml) exhibited a non-significant trend of lower values of necrotic cells (Figures 3A,C). On the other hand, treatment with LPS induced a significant decrease in the sub-G1 population of CFs exposed to sI/R, compared with untreated conditions (*p* < 0.01; Figures 3B,C).

### Protective Role of TLR4 Against Cell Death Induced by Simulated Ischemia and Simulated Ischemia/Reperfusion in Cardiac Fibroblasts

**Figure 4:**
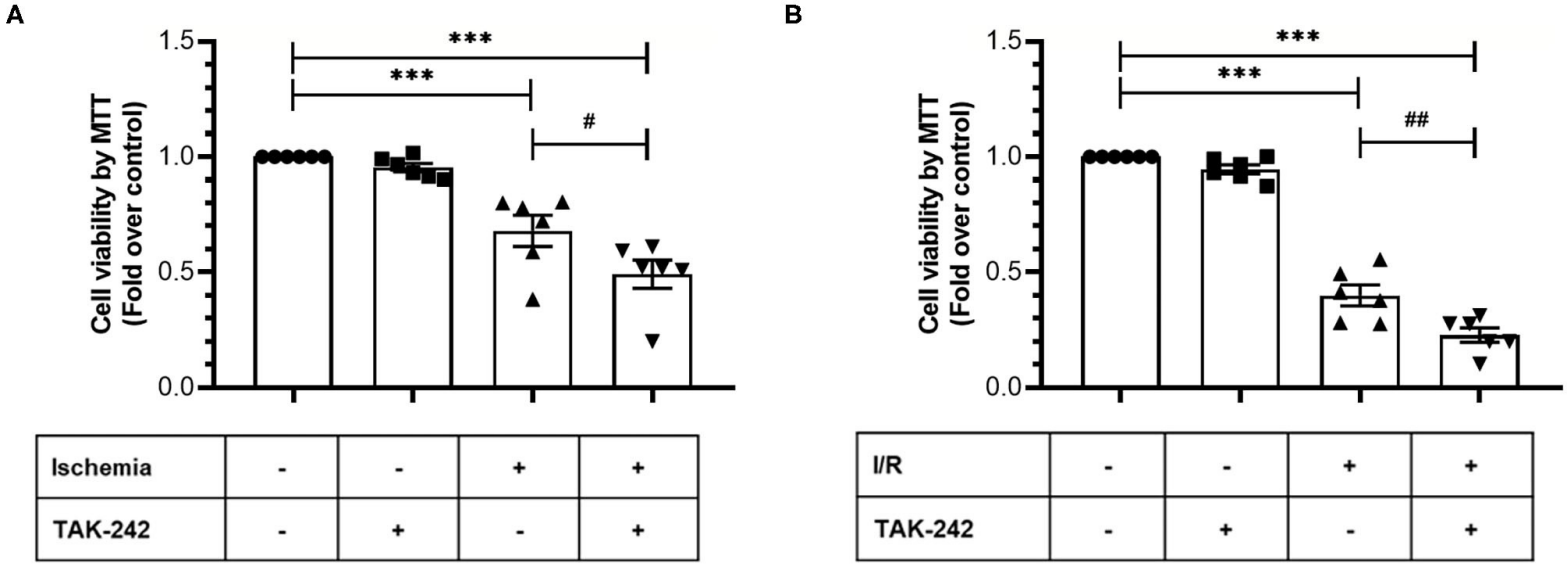
Blockade of TLR4 increases the loss of cardiac fibroblasts viability induced by ischemia or ischemia/reperfusion (I/R). Cardiac fibroblasts were treated with the specific TLR4 inhibitor TAK-242 (4 μM) during the 8 h of ischemia **(A)** or during the 8 h of ischemia and the 16 h of reperfusion **(B)**. Cell viability was determined using the MTT assay. Results are expressed as mean ± SEM of six independent experiments. ***p < 0.001 vs. control; #*p* < 0.05 vs. ischemia; ##*p* < 0.01 vs. sI/R.

**Figure 5:**
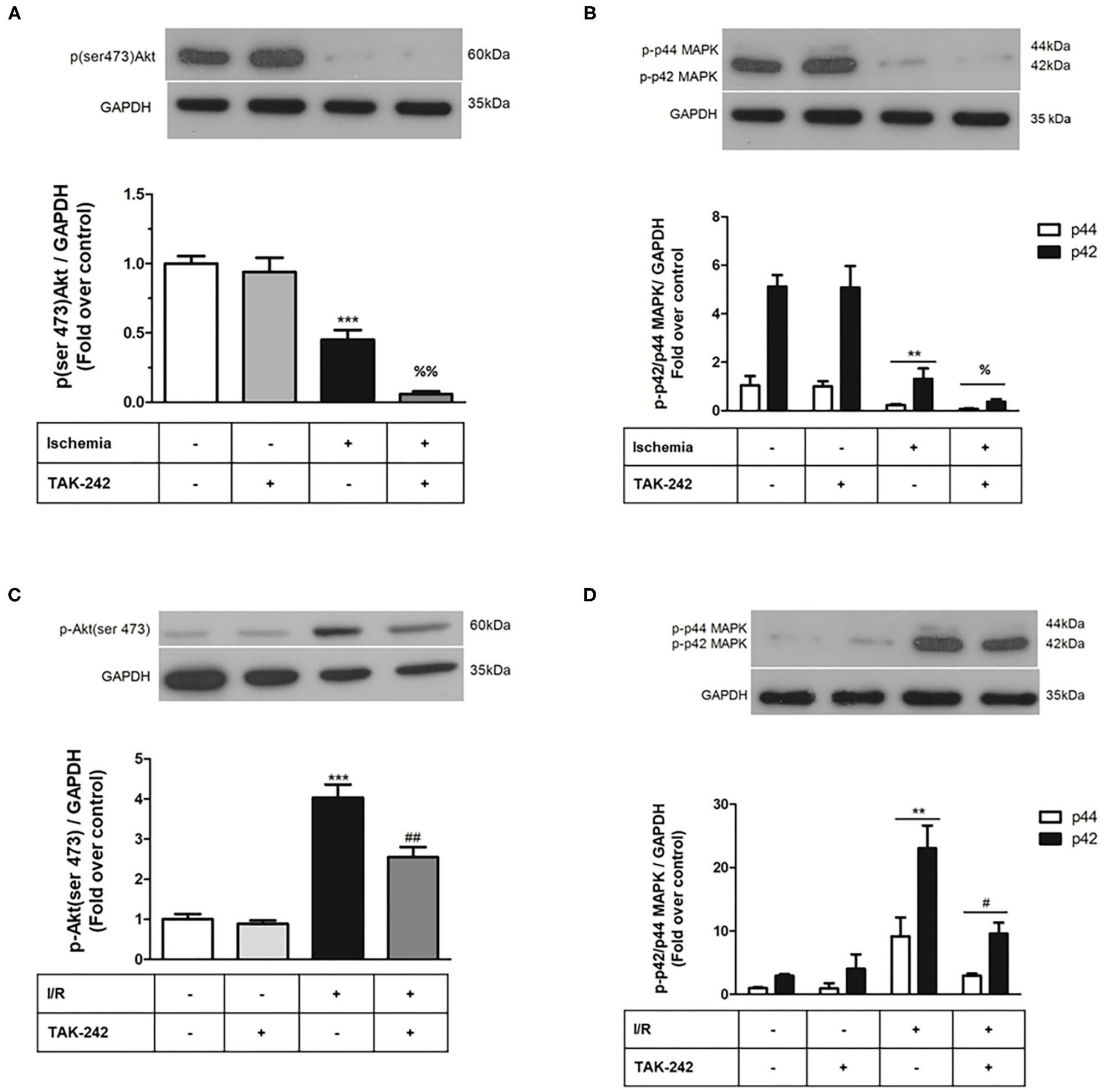
Effect of TLR4 blockade on p-ERK1/2 and p-AKT levels after treatment of cardiac fibroblasts with lipopolysaccharide (LPS) during ischemia and during ischemia and reperfusion. Cardiac fibroblasts were treated with LPS (1 μg/ml) in the presence of the TAK-242 (4 μM) inhibitor during the 8 h of ischemia **(A,B)** and during the 8 h of ischemia and the 16 h of reperfusion (sI/R) **(C,D)**. The expression levels of p-ERK1/2 and p-AKT were determined by Western blot. GAPDH was used as a loading control (we use the same blot for **A,B**). Results are expressed as mean ± SEM of four independent experiments. ***p < 0.001 vs. control; **p < 0.01 vs. control; ^%^*p* < 0.05 and ^*%%*^*p* < 0.01 vs. ischemia. ^#^*p* < 0.05 and ^*##*^*p* < 0.01 vs. sI/R.

Next, we evaluated the effects of TLR4 blockade on cell viability and intracellular survival pathways when CFs were exposed only to simulated ischemia and to sI/R. First, cell viability (measured by MTT assay) significantly decreased in CFs treated with the specific TLR4 inhibitor TAK-242 after ischemia only or sI/R, compared with the respective untreated groups (Figure 4). Furthermore, treatment of CFs with TAK-242 significantly decreased p-Akt and p-ERK1/2 protein levels (determined by western blot) in simulated ischemia only (Figures 5A,B) or sI/R (Figures 5C,D), compared with the respective untreated groups.

### The Cytoprotective Effects of Lipopolysaccharide Against Simulated Ischemia/Reperfusion-Induced Death of Cardiac Fibroblasts Are Mediated by the TLR4, Akt, and ERK1/2

**Figure 6:**
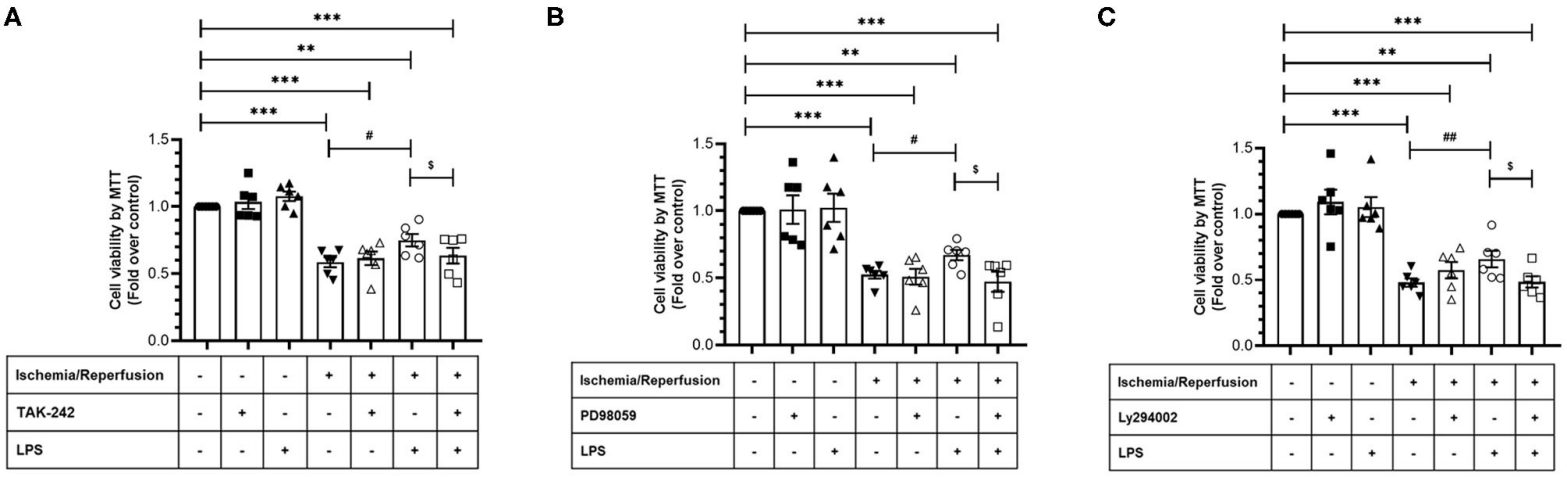
Effect of TLR4 blockade, ERK1/2, and AKT inhibition on the loss of viability induced by ischemia/reperfusion (I/R) in cardiac fibroblasts treated with lipopolysaccharide (LPS). Cardiac fibroblasts were treated with LPS (1 μg/ml) in the presence of **(A)** TAK-242 (4 μM), **(B)** PD98059 (25 μM), or **(C)** LY294002 (10 μM). Cell viability was determined by the MTT assay. Results are expressed as mean ± SEM of six independent experiments. ***p < 0.001 and **p < 0.01 vs. control; ^#^*p* < 0.05 and ^*##*^*p* < 0.01 vs. sI/R; ^$^*p* < 0.05 vs. sI/R + LPS.

After verifying the participation of the TLR4 in CF survival during sI/R, we continued with the study of the signaling pathways involved in the cytoprotective effects of LPS. To this end, we treated CFs subjected to sI/R with LPS in the presence/absence of TAK-242, LY294002 (Akt-specific inhibitor) and PD98059 (ERK1/2-specific inhibitor) and measured cell viability using the MTT assay. We found that treatments with TAK-242 (Figure 6A), LY294002 (Figure 6B), and PD98059 (Figure 6C) prevented the cytoprotective effects of LPS in CFs exposed to sI/R, compared with the respective groups without inhibitors.

## Discussion

In the present study, our main findings were as follows: (a) preconditioning or administration of LPS only in ischemia or only in reperfusion was not cytoprotective for CFs, while in CFs exposed to sI/R, LPS treatment administered during both ischemia and reperfusion increased cell viability and reduced the sub-G1 population, without reducing the number of necrotic cells; (b) in CFs subjected only to simulated ischemia or to sI/R, blockade of the TLR4 reduced cell viability and activity of pro-survival kinases Akt and ERK1/2; and (c) in CFs subjected to sI/R, blockade of the TLR4, or inhibition of the Akt or ERK1/2 signaling pathways abolished the cytoprotective effects of LPS on CF viability.

### Lipopolysaccharide Prevents Cardiac Fibroblasts Apoptosis Induced by Simulated Ischemia/Reperfusion

Our results showed that in CFs, LPS preconditioning for 24 and 16 h did not protect against the deleterious effects induced by sI/R. In contrast, several studies have demonstrated that LPS preconditioning during 24 h or longer periods produces cardioprotective effects in animals before the development of MIR (18–21). We consider that the differences with our results could be due to several factors, such as *in vivo* vs. *in vitro* models, the use of different cells types, and LPS concentration and/or exposure/administration time. In this regard, Ha et al. (18) showed in *in vivo* models of cardiac I/R that in order to be effective, TLR4 agonists must be administered at least 8–24 h prior to the induction of I/R. This has been referred to as myocardial preconditioning or tolerance, and this preconditioning effect is reached at lower TLR4 concentrations (in the order of ng/ml). Previous studies have shown that pretreatment of animals with a small dose of LPS for 24 h results in significant inhibition of NF-kB activation and protection of the myocardium against I/R injury (22–24). The authors suggest that LPS pretreatment induces a feedback regulation mechanism of IkBa expression in the myocardium and, consequently, reduces NF-KB activation. It is well-known that LPS administration increases the expression of inflammatory cytokines (such as TNF-α, IL-1β, and IL-6) in the host through the TLR4-mediated NF-kB activation pathway. Consequently, administration of a large dose of LPS rapidly induces NF-kB activation and leads to the release of inflammatory cytokines, which play a key role in cardiac dysfunction and injury (25, 26). In this sense, we have previously observed that LPS triggers an increase in cytokine and chemokine levels in CFs (15); however, our results indicated that LPS did not produce additional death of CFs at higher LPS concentrations. In addition, an interesting topic to discuss about our results is that LPS did not prevent CF viability loss triggered by I/R only during ischemia or only during reperfusion; however, when LPS was administered during both ischemia and reperfusion, CF viability increased, compared with groups without treatment. These results are similar to those observed by Chao et al. (23) in cardiomyocytes subjected to hypoxia/serum deprivation, where LPS treatment (500 ng/ml) increased cell viability in both conditions and reduced DNA fragmentation, apoptosis, and caspase-3 activation, corroborating that LPS administration during sI/R can confer cytoprotection. The lack of effect of LPS in conditions of ischemia only or reperfusion only suggests that survival kinases such as the RISK must be continuously active.

### Lipopolysaccharide Activates AKT and ERK1/2 to Reduce Cardiac Fibroblast Apoptosis Induced by Simulated Ischemia/Reperfusion

The RISK pathway corresponds to the activation of two parallel cascades: PI3K-Akt and MEK1-ERK1/2, a group of pro-survival protein kinases that confer cardioprotection when activated specifically at the time of reperfusion (20). We have previously reported in CFs that TLR4 stimulation with LPS induces activation of Akt, ERK1/2, and NF-kB signaling pathways (17). In this regard, our results showed that in CFs subjected to sI/R conditions, LPS triggers an early activation of Akt and ERK1/2 pathways, probably as a survival cell response. Moreover, our results show now that TLR4 blockade with TAK-246 greatly reduces cell viability and phosphorylation of Akt and ERK1/2 during the treatments of simulated ischemia only and sI/R only, suggesting that TLR4 activation exerts a protective role in CFs during these adverse conditions.

The cytoprotective effects of LPS treatment on CF viability subjected to sI/R were prevented when Akt or ERK1/2 were inhibited, confirming that RISK intracellular signaling is an indispensable survival pathway as a consequence of TLR4 activation by LPS. In this sense, Liu et al. (19) reported that treatment with LPS, through the PI3K/Akt pathway, conferred cardioprotection in an *in vivo* rat model of I/R performed by left coronary artery occlusion, followed by restoration of blood flow. Similarly, Ha et al. (18) reported in an *ex vivo* rat model of MIR that a 24-h pretreatment with LPS decreased the number of apoptotic cardiomyocytes and cardiac injury through an AKT-dependent signaling pathway (18). In addition, other studies have indicated that clenbuterol (potent and selective beta-2 adrenergic agonist) increases the phosphorylation of ERK1/2, thus inhibiting myocardial apoptosis, as indicated by the reduction of Bax/Bcl-2 mRNA and caspase-3 protein levels (27) and that ERK1/2 inhibition exaggerates reperfusion injury in isolated rat hearts (28). Activation of the ERK1/2 and Akt pathways in CFs under sI/R (8, 10) and other related studies (18, 28) have demonstrated the relevance of preventing apoptosis to improve healing process. In our *in vitro* model, LPS treatment showed anti-apoptotic effects with the decrease of the sub-G1 population. Anti-apoptotic effects of LPS have been reported in cardiomyocytes exposed to hypoxia/serum deprivation, in which DNA fragmentation is decreased and pro-caspase 3 cleavage is inhibited (23). With regard to signaling pathways, Chao et al. (23) showed that PI3K/Akt, ERK1/2, and IkappaB kinase beta contribute to the anti-apoptotic effect of LPS since the specific inhibitors wortmannin and PD98059, and dominant negative IKKbeta transgene expression reversed the LPS effect. Our results are reinforced by our previous findings, where we showed that TGF-β1 reduces cell viability loss triggered by I/R through a mechanism involving PI3k/AKT, ERK1/2, and SMAD protein (10). In the same line, we showed that a mixture of ascorbic acid, *N*-acetylcysteine, and deferoxamine (A/N/D) prevents CF viability loss though an AKT and ERK1/2 mechanism (11). In addition, clenbuterol—which increases ERK1/2 activation—also reduces caspase-3 expression, and AKT activation also reduces caspase-3 activation in cardiomyocytes exposed to I/R (27). Altogether, our results confirm that PI3K/Akt and ERK1/2 are pro-survival kinase pathways triggered by LPS/TLR4, suggesting TLR4 activation as a potential pharmacological target to confer CF protection in the I/R context.

In contrast, other studies have shown that TLR4 activation triggers cell death under I/R conditions. A recent report indicated that TLR4 deficiency contributes to less myocardial injury and inflammatory symptoms after the induction of I/R (29). The inflammatory reaction is a key hallmark of I/R, and its modulation has been shown to be beneficial in previous investigations (30, 31). Specifically, the TLR4/NF-κB signaling pathway plays a pivotal role in I/R injury, promoting a marked expression of proinflammatory cytokines in cardiomyocytes under I/R conditions (32). It is well-known that LPS administration increases the expression of inflammatory cytokines (such as IL-1β, IL-6, and TNF-α) in the host through the TLR4/NFkB pathway but can also induce the PI3K/Akt pathway, which has been reported as an endogenous negative feedback regulator and/or compensatory mechanism of TLR4/NFkB-mediated proinflammatory responses, thus preventing an excessive innate immune response (25, 26, 33, 34). In this sense, in our previous findings, we showed that I/R triggers the activation of p38 and JNK signaling pathways (11), which correspond to proapoptotic signals. In addition, a recent study showed that miR-20a has a protective action on cardiomyocytes subjected to sI/R by inactivating the p38 MAPK/JNK pathway and promoting cell viability (35). However, in our present study, we did not evaluate whether LPS treatment can reduce p38 and JNK activation, so this topic remains to be elucidated. Finally, several studies have shown that LPS preconditioning protects against I/R damage, although the mechanisms are quite different and depend on the animal model, tissue, or cell type involved. In this sense, He et al. (36) demonstrated that preconditioning with LPS led to the accumulation of HIF-2α in kidneys and mouse endothelial cells, as a result of the activation of NF-κB, which subsequently improved kidney microvascular perfusion and reduced ischemic tubular damage. By contrast, Yao et al. (37) showed that preconditioning with LPS at low doses resulted in significantly higher levels of HSP70 in the myocardium, which could dramatically inhibit the translocation of NF-κB and that NF-κB inhibition, in turn, attenuated the release of inflammatory cytokines (TNF-α, IL-1β, and IL-6) and reduced myocardial apoptosis and infarction area after injury by I/R. In addition, LPS preconditioning protects hepatocytes from I/R injury by inhibiting the ATF4-CHOP pathway, which may be critical in reducing apoptosis-related molecules and modulating innate inflammation (38). Along the same lines, Sano et al. (39) demonstrated that liver preconditioning with LPS caused the positive regulation of specific negative regulators in the TLR4 signaling pathway and that the pre-ischemic induction of these regulators plays an important role as an immunological preparation for subsequent I/R and produces resistance to liver injury. Finally, Merry et al. (40) demonstrated that low doses of LPS protect against subsequent pulmonary I/R injury. LPS preconditioning reduced lung injury and the production of inflammatory mediators after post-I/R exposure. These results as a whole demonstrate that the inhibition of NF-KB, rather than its activation, is an important factor in cytoprotection against I/R injury.

## Conclusions

In conclusion, our findings indicate that LPS treatment, administered in both ischemia and reperfusion, prevents loss of viability and apoptosis in CFs exposed to sI/R, through a mechanism mediated by the activation of the TLR4 and the Akt and ERK1/2 pro-survival kinases, suggesting TLR4 activation as a potential pharmacological target to prevent cell death by sI/R. We recently showed that CF death induced by antioxidant treatment maintains certain cellular functions, such as collagen secretion, CF-to-CMF differentiation, and migration, which are necessary to achieve an affective wound repair process. Therefore, our results suggest that TLR4 activation in CFs could be another effective strategy to prevent excessive cell death and improve the mechanisms associated with wound repair after myocardial infarction.

## Data Availability Statement

The raw data supporting the conclusions of this article will be made available by the authors, without undue reservation.

## Ethics Statement

The animal study was reviewed and approved by our Institutional Ethics Committee (Protocol code CBE2017-13-CYQ-UCH approved on March 14, 2018).

## Author Contributions

PP-F: experimental analyses, analysis of data, and writing of manuscript. JE-C, CE-P, and CQ: experimental analyses. FB and PA: experimental analyses and analysis of data. AS-H and VP-J: analysis of data. GD-A: conception of the research, analysis of data, and writing of the manuscript. All authors contributed to the article and approved the submitted version.

## Conflict of Interest

The authors declare that the research was conducted in the absence of any commercial or financial relationships that could be construed as a potential conflict of interest.
